# Comparison of Cultivars and Seasonal Variation in Blueberry (*Vaccinium* Species) Leaf Extract on Adult T-Cell Leukemia Cell Line Growth Suppression

**DOI:** 10.3390/medicines1010003

**Published:** 2014-06-30

**Authors:** Hisahiro Kai, Takuichi Fuse, Hisato Kunitake, Kazuhiro Morishita, Koji Matsuno

**Affiliations:** 1 Department of Pharmaceutical Health Sciences, School of Pharmaceutical Sciences, Kyushu University of Health and Welfare, 1714-1 Yoshino-machi, Nobeoka, Miyazaki 882-8508, Japan; E-Mail: kjmtsn@phoenix.ac.jp; 2 Department of Biochemistry and Applied Biosciences, Faculty of Agriculture, University of Miyazaki, 1-1 Gakuenkibanadai-nishi, Miyazaki 889-2192, Japan; E-Mails: awaki2829@yahoo.co.jp (T.F.); hkuni@cc.miyazaki-u.ac.jp (H.K.); 3 Division of Tumor and Cellular Biochemistry, Department of Medical Sciences, Faculty of Medicine, University of Miyazaki, 5200 Kihara Kiyotake, Miyazaki, Miyazaki 889-1692, Japan; E-Mail: kmorishi@fc.miyazaki-u.ac.jp

**Keywords:** adult T-cell leukemia, blueberry leaves, rabbit-eye blueberry, defoliation season, cell proliferation assay

## Abstract

The inhibitory effects of blueberry leaves on the proliferation of adult T-cell leukemia (ATL) cell lines have previously been reported. A comparison of blueberry leaf extracts from different cultivars and seasonal variation were investigated regarding their effects on ATL cell line proliferation. The inhibitory effects of 80% ethanol leaf extracts from different blueberry cultivars collected from April to December in 2006 or 2008 were evaluated using two ATL cell lines. The bioactivities of leaf extracts of rabbit-eye blueberry (*Vaccinium virgatum* Aiton; RB species), southern highbush blueberry (*V.* spp.; SB species), northern highbush blueberry (*V. corymbosum* L.; NB species), and wild blueberry (*V. bracteatum* Thunb.; WB species) were compared. Of these, leaves of the RB species collected in December showed a significantly stronger inhibitory effect in both cell lines than the SB, NB, or WB species. These results suggest elevated biosynthesis of ATL-preventative bioactive compounds in the leaves of the RB species before the defoliation season.

## 1. Introduction

Adult T-cell leukemia (ATL) occurs in a small population of individuals infected with human T-cell leukemia virus type I (HTLV-I). After infection with HTLV-I, 2% to 5% of carriers are likely to develop ATL after a long latency period of 30 to 50 years [1]. Affected patients have frequently been identified to be from specific tropical regions [2]. ATL has a poor prognosis, with a mean survival time of 13 months, being refractory to currently available combination chemotherapy [3]. Therefore, it is important to continue the search for an appropriate therapeutic method to prevent the development of ATL or to prolong survival after its occurrence. A previous paper reported the results of a screening test of 52 samples of agricultural plants for their ability to inhibit proliferation in seven ATL-related cell lines, with blueberry leaves showing significant inhibitory effects [4].

The leaves of blueberry, which belongs to the Ericaceae family, have been used as an anti-diabetic folk medicine in Europe and Canada [5,6]. There are four species of blueberry: rabbit-eye blueberry (*Vaccinium virgatum* Aiton: RB species), southern highbush blueberry (*V.* spp.: SB species), northern highbush blueberry (*V. corymbosum* L.: NB species) and wild blueberry (*V. bracteatum* Thunb.: WB species), with several cultivars existing in each type. These species differ distinctly in cold resistance, height and fruit size [7]. It is important to clarify the effects of differences in blueberry cultivars and seasonal variation on ATL cell growth suppression. In the previous paper [4], ATL cell proliferation was assessed using a leaf extract from the Homebell cultivar of the RB species. However, there are no reports of detailed investigations regarding differences among the multitude of blueberry cultivars. Such studies could reveal information on potential material for use in the manufacturing of natural medicines or functional foods. In the present study, various blueberry cultivars were screened and assessed for the effects of seasonal variation on their ability to inhibit the proliferation of ATL cell lines with the aim of identifying the optimal cultivars and collection times for use in the prevention and treatment of ATL.

## 2. Experimental Section

### 2.1. Plant Materials

All blueberry cultivars used were cultivated in Miyazaki, Japan. All cultivars were taxonomically identified on the basis of morphological characteristics, and voucher specimens were deposited at the University of Miyazaki. The voucher numbers are provided in Table 1 and Table 2. In 2006, fresh leaves of the RB species (Homebell, Myers and Tifblue) were collected every month from April to December (Table 1). In 2008, the leaves of 20 cultivars of blueberry were also collected every two months from April to December (Table 2). In Japan, the blueberry drops its leaves during January to March. The 11 cultivars are as listed in the Results and Discussion (Section 3.2).

**Table 1:** The yield of 80% ethanol extracts from blueberry leaves collected in 2006.

Species	Cultivars	*Voucher Number* Yield (%)								
		April	May	June	July	August	September	October	November	December
RB	Homebell	*RB121*	*RB122*	*RB123*	*RB124*	*RB125*	*RB126*	*RB127*	*RB128*	*RB129*
		48.7%	37.1%	40.5%	37.1%	39.3%	43.9%	40.3%	44.7%	41.2%
	Myers	*RB131*	*RB132*	*RB133*	*RB134*	*RB135*	*RB136*	*RB137*	*RB138*	*RB139*
		43.3%	43.2%	43.7%	45.7%	44.9%	43.3%	41.9%	42.5%	37.0%
	Tifblue	*RB141*	*RB142*	*RB143*	*RB144*	*RB145*	*RB146*	*RB147*	*RB148*	*RB149*
		39.6%	33.2%	38.5%	30.8%	36.7%	35.2%	34.5%	37.0%	34.2%

### 2.2. Extraction

The fresh blueberry leaves were freeze-dried and powdered. The freeze-dried powders (approximately 10 mg) were extracted with 300 μL of 80% ethanol (50 °C, 1 h). The extracts were filtered using a centrifugal filter (Cosmospin Filter H 0.45 μm, Nacalai, Kyoto, Japan) (3000 rpm × 5 min). The filtrate was dried, dissolved in dimethyl sulfoxide, and diluted in RPMI 1640 medium prior to utilization in ATL cell proliferation assays. These vehicles did not affect the cytotoxicity of ATL cell lines from our preliminary experiments. The yields of the 80% ethanol extracts (% yield = (80% ethanol extracts (mg)/freeze dried powder (mg)) × 100) are presented in Table 1 and Table 2.

**Table 2:** The yield of 80% ethanol extracts from blueberry leaves collected in 2008.

Species	Cultivars	*Voucher Number* Yield (%)				
		April	June	August	October	December
RB	Climax	*RB011*	*RB012*	*RB013*	*RB014*	*RB015*
		28.8%	34.7%	42.0%	44.5%	37.7%
	Suwannee	*RB021*	*RB022*	*RB023*	*RB024*	*RB025*
		35.6%	35.0%	35.5%	34.2%	41.6%
	Myers	*RB031*	*RB032*	*RB033*	*RB034*	*RB035*
		34.1%	42.8%	38.5%	40.9%	41.6%
	Callaway	*RB041*	*RB042*	*RB043*	*RB044*	*RB045*
		37.3%	39.1%	39.9%	39.4%	41.9%
	Ethel	*RB051*	*RB052*	*RB053*	*RB054*	*RB055*
		32.0%	40.1%	43.4%	39.9%	34.5%
	Southland	*RB061*	*RB062*	*RB063*	*RB064*	*RB065*
		25.4%	40.6%	40.9%	41.3%	35.5%
	Bluebelle	*RB071*	*RB072*	*RB073*	*RB074*	*RB075*
		28.7%	37.1%	41.2%	40.6%	43.5%
	Gardenblue	*RB081*	*RB082*	*RB083*	*RB084*	*RB085*
		36.5%	46.4%	41.5%	42.0%	38.4%
	Woodard	*RB091*	*RB092*	*RB093*	*RB094*	*RB095*
		38.2%	41.9%	43.0%	40.0%	31.1%
	Red Pearl	*RB101*	*RB102*	*RB103*	*RB104*	*RB105*
		24.2%	40.5%	47.0%	43.3%	44.2%
	Homebell	*RB111*	*RB112*	*RB113*	*RB114*	*RB115*
		39.5%	35.7%	34.6%	42.3%	38.9%
SB	O’Neal	*SB011*	*SB012*	*SB013*	*SB014*	*SB015*
		25.6%	34.4%	32.6%	32.3%	26.5%
	Reveille	*SB021*	*SB022*	*SB023*	*SB024*	*SB025*
		34.2%	33.1%	36.0%	39.0%	31.2%
	Sunshineblue	*SB031*	*SB032*	*SB033*	*SB034*	*SB035*
		36.5%	32.4%	35.9%	37.0%	34.2%
	Sharpblue	*SB041*	*SB042*	*SB043*	*SB044*	*SB045*
		33.7%	31.0%	33.8%	32.7%	32.9%
	Flodablue	*SB051*	*SB052*	*SB053*	*SB054*	*SB055*
		31.1%	29.4%	32.0%	32.7%	32.0%
NB	Spartan	*NB011*	*NB012*	*NB013*	*NB014*	*NB015*
		32.7%	27.9%	21.4%	34.9%	24.3%
	Bluecrop	*NB021*	*NB022*	*NB023*	*NB024*	*NB025*
		19.0%	25.2%	29.9%	32.5%	22.1%
	Berkeley	*NB031*	*NB032*	*NB033*	*NB034*	*NB035*
		25.1%	33.7%	32.3%	32.7%	30.7%
WB	Shashanbo	*WB011*	*WB012*	*WB013*	*WB014*	*WB015*
		13.9%	32.9%	26.4%	34.3%	33.5%

### 2.3. Cell Culture

In this study, two ATL cell lines (ED and Su9T01), which are highly responsive to blueberry extracts based on previous results [4], were used. ED and Su9T01 cells were kindly provided by Dr. M. Maeda (Kyoto University, Kyoto, Japan) and Dr. N. Arima (Kagoshima University, Kagoshima, Japan), respectively. All cells were maintained in RPMI 1640 medium (Sigma-Aldrich Co., St. Louis, MO, USA) supplemented with 10% foetal bovine serum (SAFC Biosciences, Lenexa, KS, USA, Lot No. 3M0469) containing 100 U mL^−1^ penicillin G and 100 μg mL^−1^ streptomycin (Sigma-Aldrich Co., St. Louis, MO, USA). Each cell line was subcultured twice a week, and cell numbers were adjusted to 1 × 10^5^ cells mL^−1^ for the *in vitro* experiments.

### 2.4. Cell Proliferation Assay

Each cell line was seeded (1 × 10^5^ cells mL^−1^, 90 μL per well) into a 96-well plate containing RPMI 1640 medium. After incubation at 37 °C for 24 h in an atmosphere containing 5% CO_2_, the blueberry extracts were added (10 μL per well) to the cells and incubated for an additional 72 h. Subsequently, the inhibition of cell proliferation was determined using a 2-(2-methoxy-4-nitrophenyl)-3-(4-nitrophenyl)-5-(2,4-disulfophenyl)-2*H*-tetrazolium monosodium salt (WST-8) assay kit (Dojindo, Kumamoto, Japan). Viable cells convert the tetrazolium salt in WST-8 to the highly water-soluble formazan, which was monitored by measuring absorbance at 450 nm with a microplate reader (Wallac 1420 ARVOsx, Perkin-Elmer Japan, Yokohama, Japan). The sample results are presented as relative to control (*i.e.*, % control = (formazan dye of the sample group/formazan dye of the control group) × 100). Genistein (Wako, Osaka, Japan) was used as a positive control [8,9].

### 2.5. Statistical Analysis

Data are expressed as mean ± SD (*n* = 3). Statistical differences between the sample and genistein (positive control) groups were evaluated by analysis of variance (ANOVA) followed by Dunnett’s *post-hoc* test. Values with *p* < 0.05 were considered to be significant.

## 3. Results and Discussion

### 3.1. Seasonal Variation in Cell Inhibition of 80% Ethanol Extracts from Leaves of the Rabbit-Eye Blueberry Species (Vaccinium virgatum Aiton; RB Species) Collected in 2006

**Figure 1:**
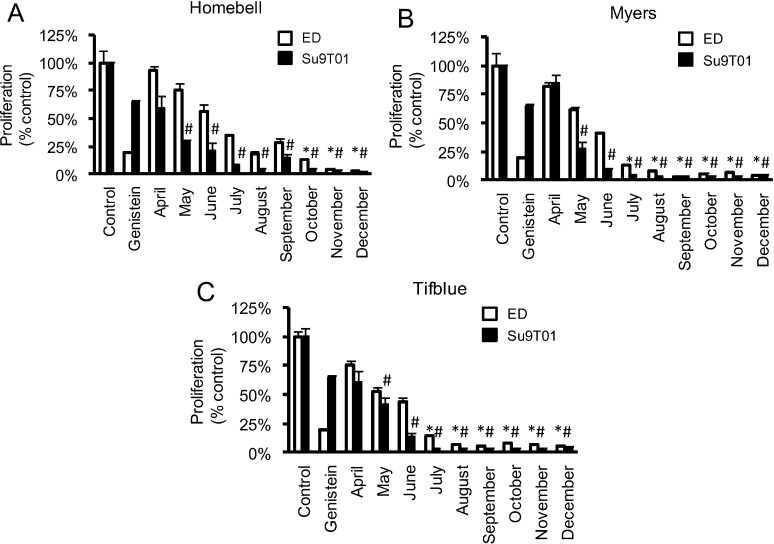
Seasonal variation in cell inhibition of 80% ethanol extracts from leaves of the rabbit-eye blueberry species (*Vaccinium virgatum* Aiton; RB species) collected in 2006 on two ATL cell lines, ED (□) and Su9T01 (■).

Initially, seasonal variation in inhibitory effects on ED and Su9T01 cell proliferation was assessed using extracts from the leaves (50 μg mL^−1^) of three cultivars of the RB species (Homebell, Myers and Tifblue), collected every month from April to December in 2006. Leaves were not collected during the fall season (from January to March). Among these cultivars, Homebell (50 μg mL^−1^), collected from October to December, showed significantly greater ED cell inhibition than genistein (50 μM), whereas from May to December, this cultivar (50 μg mL^−1^) showed significantly greater Su9T01 cell inhibition than genistein (50 μM) (Figure 1A). Both Myers and Tifblue (50 μg mL^−1^), collected from July to December, showed significantly greater ED cell inhibition than genistein (50 μM), whereas from May to December, it showed significantly greater Su9T01 cell inhibition than genistein (50 μM) (Figure 1B,C). These results indicate that the inhibitory activity in leaf extracts of the RB species tends to increase as the defoliation season approaches, supporting the previous report [4].

Each column represents the mean ± SD of three independent tests. Significant differences between the sample and 50 μM genistein (positive control) groups are indicated as follows: * *p* < 0.05 for ED and # *p* < 0.05 for Su9T01 (ANOVA followed by Dunnett’s *post-hoc* test).

### 3.2. Comparison of Cultivars and Seasonal Variation in the Extracts of Blueberry Leaves Collected in 2008 on the Suppression of ATL Cell Growth

**Figure 2:**
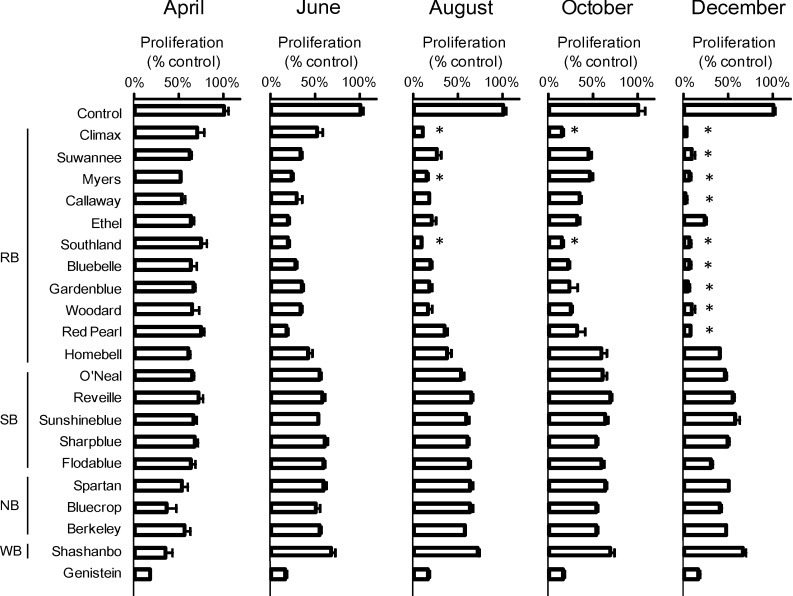
Comparison of cultivars and seasonal variation in the extracts of blueberry leaves collected in 2008 on the suppression of ED cell growth.

**Figure 3:**
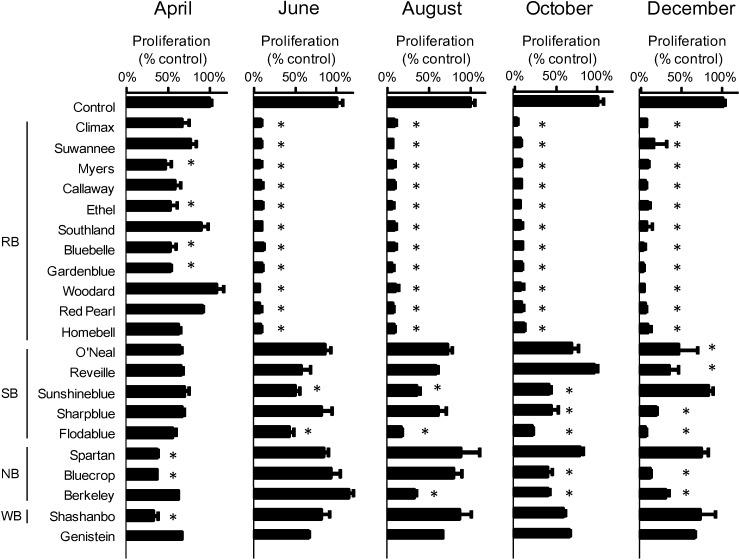
Comparison of cultivars and seasonal variation in the extracts of blueberry leaves collected in 2008 on the suppression of Su9T01 cell growth.

Next, a more extensive comparison of cultivars was undertaken with cultivars of other species collected in 2008 regarding seasonal variation in RB extract inhibition of ATL cell growth. Twenty cultivars were collected over a five-month period. These cultivars included 11 cultivars of the RB species (Climax, Suwannee, Myers, Callaway, Ethel, Southland, Bluebelle, Gardenblue, Woodard, Red Pearl and Homebell), five cultivars of the SB species (O’Neal, Reveille, Sunshineblue, Sharpblue, Flodablue), three cultivars of the NB species (Spartan, Bluecrop, Berkeley) and one cultivar of the WB species (Shashanbo). Unfortunately, due to poor growing conditions in 2008, Tifblue could not be collected. In ED cells, the RB species (except for Ethel and Homebell) from December (50 μg mL^−1^) showed greater inhibitory activity than genistein (50 μM) (Figure 2). In particular, Climax and Southland in August and October (50 μg mL^−1^) showed a significantly greater inhibitory effect than genistein (50 μM) in ED cells. In Su9T01 cells, all cultivars of the RB species showed markedly greater inhibitory activity from June to December (50 μg mL^−1^) than genistein (50 μM) (Figure 3).

Each column represents the mean ± SD of three independent tests. Significant differences between the sample and 50 μM genistein (positive control) groups are indicated as follows: * *p* < 0.05 (ANOVA followed by Dunnett’s *post-hoc* test).

Each column represents the mean ± SD of three independent tests. Significant differences between the sample and 50 μM genistein (positive control) groups are indicated as follows: * *p* < 0.05 (ANOVA followed by Dunnett’s *post-hoc* test).

Furthermore, Myers, Ethel, Bluebelle and Gardenblue showed a significantly greater inhibitory effect in April (50 μg mL^−1^) than genistein (50 μM) in Su9T01 cells, which is in contrast to observations with ED cells. With respect to Su9T01 cell inhibition by the SB species, inhibitory effects were seen in the December samples of O’Neal and Reveille, the June to October samples of Sunshineblue, the October to December samples of Sharpblue, and the June to December samples of Flodablue. Regarding Su9T01 cell inhibition by the NB species, inhibitory effects were seen only in the April sample of Spartan, the April, October and December samples of Bluecrop, and the August to December samples of Berkeley. The WB species (Shashanbo) showed an inhibitory effect only in the April sample. Thus, samples from the SB, NB and WB species (50 μg mL^−1^) exhibited only a partially greater inhibitory effect than genistein (50 μM) in Su9T01 cells, unlike that observed with ED cells.

In a previous report [4], an 80% ethanol extract from leaves of the Homebell cultivar (RB species) collected in August 2004 was used. The present cultivar comparison of blueberry leaf extracts indicates that other cultivars of the RB species also appear to markedly inhibit ATL cell proliferation. With respect to seasonal variation, extracts from the RB species collected closer to December tended to exhibit greater ATL cell inhibition than other species. These trends suggest that the RB species is the most likely to contain active compounds. Genistein showed greater inhibition of ED cells than Su9T01 cells, whereas the RB species clearly showed greater inhibition of Su9T01 cells than ED cells.

Matsuo *et al.* reported that the constituents of leaves of the RB species were mainly proanthocyanidins (PAs) [10]. PAs, also known as condensed tannins, are oligomeric or polymeric products of the flavonoid (including anthocyanidin, catechin and catechin gallate ester groups) biosynthetic pathway. These compounds have been reported to exhibit a wide range of biological activities [11]. Li *et al.* reported that epigallocatechin-3-gallate inhibits proliferation of ATL, as well as HTLV-I-infected cells, by suppressing HTLV-I pX gene expression and inducing apoptotic cell death [12]. From these reports, PAs from the leaves of the RB species contribute to the specific inhibitory effect on Su9T01 cells proliferation via a similar mechanism.

## 4. Conclusions

The leaves of the RB species collected in December were the most effective inhibitors of proliferation in ATL cell lines. Thus, the best time to collect leaves of the RB species is before defoliation. This is the first study to extensively analyze differences in blueberry leaf extracts according to cultivar comparison and seasonal variation utilizing screening of ATL cell proliferation. Identification of the novel active compounds in blueberry leaves for use in the treatment and prevention of ATL will be undertaken in the future.
